# Factors associated with acceleration of clinical development for infectious diseases: a cross-sectional analysis of 10-year EMA registration data

**DOI:** 10.1016/j.lanepe.2024.100983

**Published:** 2024-06-24

**Authors:** Hanna K. de Jong, Sabine M. Hermans, Sophie M. Schuitenmaker, Maya Oli, Mariëtte A. van den Hoven, Martin P. Grobusch

**Affiliations:** a Amsterdam UMC, Location University of Amsterdam, Center for Tropical Medicine and Travel Medicine, Department of Infectious Diseases, Amsterdam Public Health – Global Health, and Amsterdam Institute for Immunology and Infectious Diseases, Amsterdam, the Netherlands; b Amsterdam UMC, Location University of Amsterdam, Department of Global Health, Amsterdam Institute for Global Health and Development, Amsterdam, the Netherlands; c Amsterdam UMC, Location VU University Amsterdam, Department of Ethics, Law and Humanities, Amsterdam, the Netherlands; d Institute of Tropical Medicine, German Centre for Infection Research (DZIF), University of Tübingen, Tübingen, Germany; e Centre de Recherches Médicales en Lambaréné (CERMEL), Lambaréné, Gabon; f Masanga Medical Research Unit (MMRU), Masanga, Sierra Leone; g Institute of Infectious Diseases and Molecular Medicine (IDM), University of Cape Town, Cape Town, South Africa

**Keywords:** Clinical trials, Drug and vaccine development, Acceleration, Infectious diseases, COVID-19

## Abstract

**Background:**

Clinical trials feature centrally in the development of drugs and vaccines to determine safety and efficacy. Clinical development can be slow and may have a duration of more than ten years. Global public health threats such as Ebola virus disease (EVD) and COVID-19 have demonstrated that it is possible to accelerate clinical trials while maintaining safety and efficacy. We investigated acceleration in clinical trials over the past decade and identified factors associated with acceleration for drugs targeting infectious diseases.

**Methods:**

A cross-sectional study was performed of all medicinal compounds targeting infectious diseases that received marketing authorisation by the European Medicines Agency (EMA) between 2012 and 2022. We calculated median clinical development time in years between the first phase 1 trial enrolment date and the authorisation date. Multivariable linear regression analysis was performed to identify factors associated with shorter development times.

**Findings:**

Eighty-one trajectories were included. The median clinical development time was 7.3 years (IQR 4.4–12.3). The fastest times belonged to drugs and vaccines targeting COVID-19 (1.3 years, IQR 0.8–1.6), EVD (5.5 years, IQR 5.1–5.8), and Hepatitis A-E (5.5 years, IQR 3.9–8.2). Factors associated with shorter development times were outbreak setting (−5.4 years [95% CI, −8.2 to −2.6]), accelerated assessment status (−4.0 years [95% CI, −7.6 to −0.5]), and drugs with combined compounds (−2.7 years [95% CI, −4.9 to −0.4]).

**Interpretation:**

Clinical development time for infectious disease-related drugs and vaccines was relatively short, and outbreak setting and accelerated EMA assessment were associated with shorter development times.

**Funding:**

Amsterdam Public Health research institute.

**Research in context**

**Evidence before this study**

Much has been written on clinical development of medicines and its acceleration during the COVID-19 pandemic. Key factors contributing to this acceleration are widely established and entail previous scientific innovations, political priority and public demand, unprecedented financial investment, and expedited regulatory reviews. However, in order to translate this to clinical development timelines and acceleration of drugs and vaccines targeting infectious diseases outside of a pandemic, we searched the literature for studies quantifying clinical development time using PubMed for peer reviewed papers published up to 10 Jan 2023, using the terms “Clinical development time”, “Clinical cycle time”, “Clinical trial time”, “Acceleration”, and “Shortening drug development”. The total median development time of phase 2 and phase 3 trials increased over the past decade for all pharmacotherapeutics. Multiple reasons were given such as shifting trial design strategies and increasing complexity and scale of phase 2 studies. A statistical assessment of drivers responsible for increases or decreases in clinical development times showed that trials of large molecules (such as monoclonal antibodies) were more time consuming than trials for traditional small-molecule drugs. However, regulatory tools such as expedited pathways used by regulatory bodies could reduce clinical development times for innovative drugs significantly. Notably, there were no studies quantifying clinical development times for drugs specifically targeting infectious diseases.

**Added value of this study**

This cross-sectional study offers a comprehensive analysis of the average clinical development time of infectious disease related medicines registered by the European Medicines Agency (EMA) between 2012 and 2022. By quantifying the clinical development time of 81 compounds we were able to identify factors associated with shorter clinical development times. In order to determine the influence of the COVID-19 pandemic, we performed a sensitivity analysis excluding those compounds. From the included compounds in our analysis a secondary analysis explored the differences with development timelines of the U.S. Food and Drug Administration (FDA), to allow comparison across agencies.

**Implications of all the available evidence**

Over the past decade clinical development times for drugs and vaccines targeting infectious diseases have been shorter than average, even when excluding COVID-19 targeting compounds. In our analysis, factors associated with the acceleration of clinical development times in the field of infectious diseases were mostly regulatory and political factors. Efforts to further accelerate clinical development for infectious disease compounds outside an infectious disease outbreak should focus on modifiable factors driving this acceleration such as enhancement of regulatory tools and clinical trial design. However, detailed understanding of all beneficial factors driving sustainable acceleration is required.

## Introduction

Clinical trials feature centrally in the development of both vaccines and drugs, providing the necessary data to determine the safety and efficacy of potential treatment or prophylaxis in humans.^1^ However, traditional clinical trial processes can be slow, with the average clinical developmental timelines varying greatly in the literature, estimated as ranging from 10 to 15 years from the start of a clinical trial trajectory until regulatory approval.^2^, ^3^, ^4^, ^5^

The process of developing a new medical compound begins with preclinical studies in non-human test models, and increasingly so in organoid models. Once preclinical data are gathered which are deemed sufficient to judge a potential compound safe to progress towards exposure of humans, clinical development follows. A clinical trial trajectory typically consists of three phases until registration (or marketing authorisation) and subsequently marketing, with a fourth phase being post-registration.^6^ The trials become more complex and time-consuming with subsequent phases and when targeting larger molecules (such as monoclonal antibodies), and multiple trials in each phase are often needed for regulatory approval.^5^ Mixed forms of the latter trial phases exist. Large-scale production investments are made ahead of regulatory approval, as the commercial product (including the manufacturing) are part of the registration and approval package. Phase 4 trials are carried out as post-marketing surveillance and are sometimes as highly structured as phase 3 trials.^6^ Regardless of attempts made by industry, academia, and other partners to reduce clinical development times, these continued to trend upwards over the past twenty years.^5^ In particular, the duration of phase 2 and 3 trials have increased due to both augmented scale and complexity (including protocols), which contribute a fair part to the duration and cost of new products. However, multiple novel approaches in the attempt to de-convolute, simplify and economize complex trial designs have been tried out, which have led to the introduction of multi-arm, multi-stage (MAMS) adaptive platforms, and more recently to the decentralisation of clinical trials.^7^, ^8^, ^9^

**Fig. 1:**
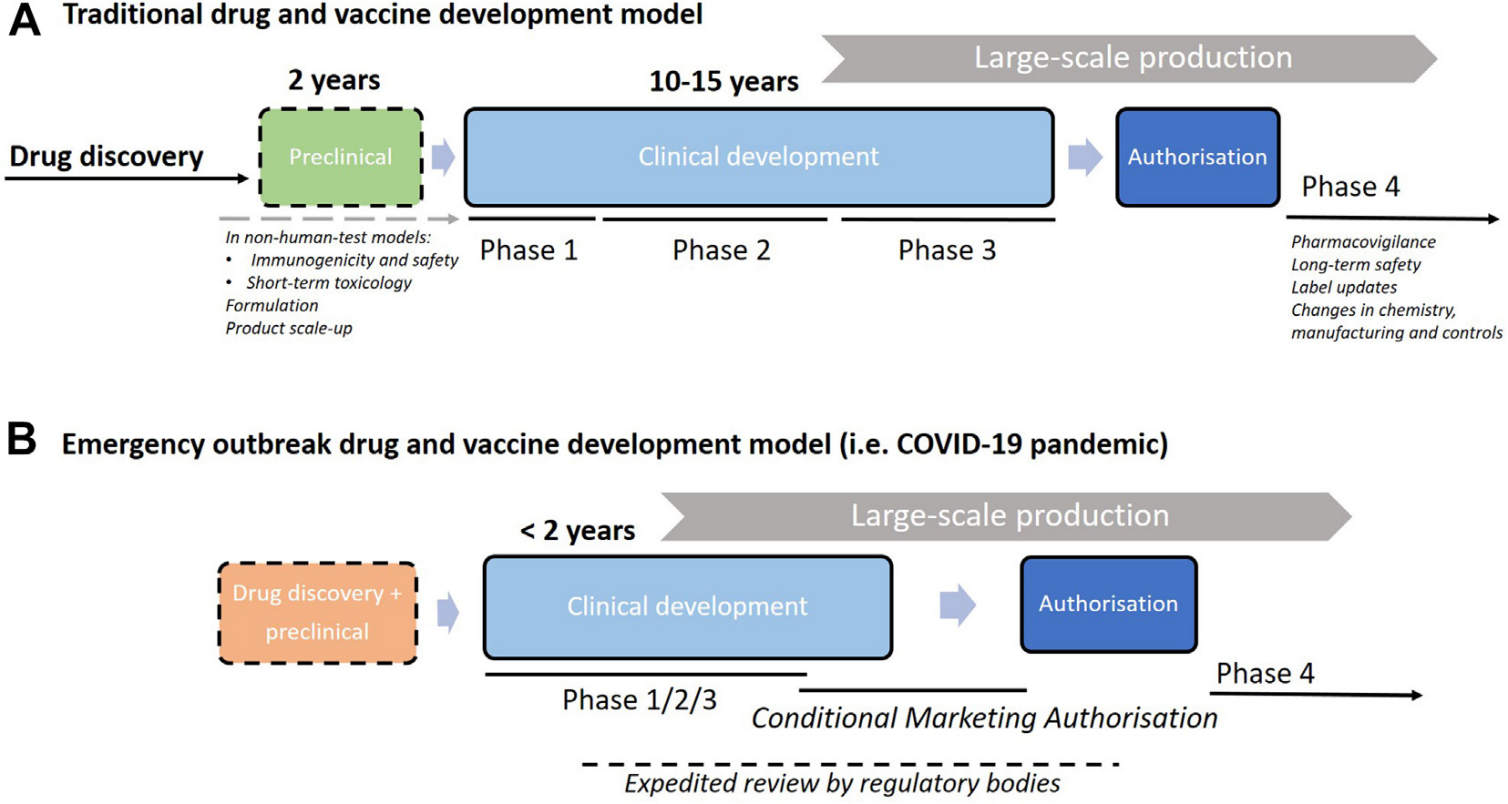
**Drug and vaccine development model**. Conceptual representation of the traditional drug and vaccine development and timeline (A) compared to the process during an emergency outbreak (B; not to scale). Drug discovery entails target selection, finding, optimization, and profiling. After this, preclinical development can start using non-human test models. The clinical development phases are subdivided in phase 1 to 3 and have their specific objectives. After phase 3, application for a new drug approval can be filed with a regulatory body. After regulatory approval or licensure, phase 4 studies can be commenced for pharmacovigilance and long-term safety. In an emergency outbreak setting (Figure 1B), drug discovery and preclinical studies (either new or commenced before the outbreak) are rapidly undertaken and large financial investments are made to jump-start clinical development. During the clinical development period, phases may be combined or overlap and expedited reviews (or rolling review) can be applied for. Conditional marketing authorisation may be granted if the benefit-risk balance of the medicine is positive and it is likely that the applicant will be able to provide comprehensive data post-authorisation, the medicine fulfils an unmet medical need, and the benefit of the medicine's immediate availability to patients is greater than the risk inherent to the fact that additional data are still required.^12^^,^^13^

Furthermore, the COVID-19 pandemic and some earlier global public health threats have demonstrated that it is possible to expedite the clinical trials of both vaccines and drugs, and that these accelerated trials may exhibit safety profiles comparable to traditional ones.^10^ The entire process of conducting clinical trials and obtaining regulatory approval for a vaccine or drug would have typically taken at least a decade, yet the first COVID-19 vaccines were developed within two years.^11^ Fig. 1 schematically shows the traditional drug and vaccine development model (Fig. 1A) and the expedited model adopted during the COVID-19 drug and vaccine development (Fig. 1b).^12^^,^^13^

The accelerated development model depicted in Fig. 1B is an example of a scenario where access to resources was unlimited and drug and vaccine development was given top priority.^14^ However, there are examples from the past where accelerations of clinical trials have occurred that were comparable in some respects to the COVID-19 pandemic. During the West African Ebola Virus Disease (EVD) outbreak in 2013–2016, several EVD vaccines were trialled in tightly connected consecutive phase 1 and 2 trials and underwent accelerated review by regulatory bodies.^15^ Another example is when drug-resistant tuberculosis (XDR-TB) was considered a serious emerging threat to public health in 2006; and consequently, bedaquiline (Sirturo®) and delamanid (Deltyba®) were accelerated through the final phases of clinical development and rapidly brought to market for a tightly selected patient population.^16^^,^^17^ This was achieved by ‘skipping’ phase 3 trials, as these were not feasible to be accomplished within a reasonable timeframe; instead, phase 4 studies were required to achieve access to a wider patient population (therefore acceleration was only limited).

When looking at these examples of acceleration, the question arises whether safety is still fully warranted, and if so, if it is justifiable to only accelerate clinical trials in the case of an emergency. Sustainably accelerating development of drugs and vaccines would increase timely accessibility to pharmaceutical development; and allow for new treatments and prophylactics to reach the public sooner. However, the concentrated efforts during COVID-19 and also EVD cannot be routinely repeated in a sustainable way due to the extreme efforts and overburdening of professionals (site teams, trialists, contract research organisations, regulatory agencies) involved. Furthermore, accelerated clinical development requires higher ‘at risk’ investments (which during COVID-19 were made possible due to the immense amount of public funding almost instantly made available); decisions to progress into larger trials will be made based on limited data, especially when moving from phase 2 to phase 3 and scale up of the manufacturing to support registration. In order to reduce risks, early phase activities need to be designed in such a way that allow for this rapid decision-making, which can be quite challenging as it involves the buying-in of all stakeholders. As a result, drugs that address diseases with a high medical need, including those associated with poverty and with a lower marketing revenue would be more likely to be developed and released onto the market.^18^

Successful examples of accelerated trials can be used to identify key factors that contributed to rapid clinical development. The commonalities among the examples given are, for instance, that they were brought to market during an emergency outbreak of an emerging infectious disease (EID), and that regulatory bodies made use of alternative or expedited regulatory pathways to try and speed up the process.^19^ For example, medicines could be granted a special status such as conditional marketing authorisation (CMA), accelerated assessment, and orphan drug designation by the European Medicine Agency (EMA) to accelerate the development and review processes.

Brown et al. (2022) published a study on clinical development times for innovative drugs and calculated the effect size of U.S. Food and Drug Administration (FDA) regulatory factors on shortening or increasing clinical development times.3 This study showed that accelerated approval, which allows for earlier approval of drugs that treat serious conditions and respond to an unmet medical need based on a surrogate endpoints granted by the FDA, was associated with a shortened clinical development time by 1.5–4.5 years. Key factors driving acceleration of clinical development time, or at least positively affecting the efficient conduct of trials, are well established in literature.^3^^,^^11^^,^^12^^,^^14^^,^^20^^,^^21^ However, most studies lack a quantitative assessment on these drivers and the actual reduction of clinical development time they would generate. Therefore the aim of this study was to calculate the current average clinical development time of infectious disease related medicines between 2012 and 2022, and to identify factors associated with shorter clinical development times. In order to determine the influence of the COVID-19 pandemic, we performed a sensitivity analysis excluding those compounds. Of included compounds we also calculated the clinical development time to registration by the FDA, to allow comparison across agencies.

## Methods

### Study design and inclusion criteria

This was a cross-sectional study of all human medicinal compounds, both drugs and vaccines, that targeted infectious diseases caused by viruses, bacteria, parasites or fungi, that were authorized by the EMA between 2012 and 2022. It was decided to only study the marketing authorisation given by the EMA and no other international regulatory bodies, as requirements for marketing authorisation could differ per regulatory body attached to different geographical locations (i.e., Europe, North America, Australia etc.). At least since 2008, all clinical trials must be registered in a database that is open to the public according to the revised Declaration of Helsinki to enhance data transparency.^22^ To increase the likelihood of obtaining the most complete dataset, the most recent timeframe of ten years after the revision of the Declaration was chosen. Generic medicines were excluded as they use the same chemical entity as a drug that has been approved earlier, and the regulatory pathways for generic drugs are significantly abridged.

### Definitions

We defined the clinical development time as the time in years between the start date of enrolment of the first in-human trial and the date of registration of the medicinal compound.

### Data sources and management

The EMA is a regulatory body that was established in 1995. It keeps an updated Excel table on its website containing all human and veterinary medicine that received a European Public Assessment Report (EPAR) and were authorized in the European Union (EU) dating back to 1996. An EPAR provides a summary of the scientific assessment of the product and is created once a medicinal product has undergone a thorough assessment by the EMA's scientific committees and working parties, and a marketing authorisation has been granted.^23^ The EMA EPAR database was accessed on 12 September 2022, and ineligible compounds were excluded. Medicines and marketing authorisation dates of the table of all EPAR's for infectious disease related medicine in humans were used to construct a new database.

As the EPAR database did not include the start dates of the first-in-human clinical trials, these were collected by two independent investigators (SS, MO) via a 3-step process between November and December 2022.

1. Start dates were gathered by searching the clinical trial registries from ClinicalTrials.gov, the EU Clinical Trials Register, and the World Health Organisation (WHO) Clinical Trial Registry for phase 1 registries.
2. If results were not available in any of these registries, the next step was by contacting the EMA, and/or the pharmaceutical companies, and/or the corresponding authors of publications on the first phase 1 studies in humans if the date was not documented in the article itself.
3. If both step 1 and 2 did not result in a start date, the search continued by going through the full public assessment report from the EMA, the medical reviews from the drug approval packages of the FDA, the Australian Public Assessment Reports (AusPAR) and the Therapeutic Goods Administration (TGA) and extensive searching through various other sources (PubMed, abstracts from conferences, websites of pharmaceutical companies).

When start dates were initially not found or were unclear, a senior researcher (HdJ) cross-checked the information obtained. If, after cross-checking, it was still not possible to retrieve an exact start date from official documents, or when uncertainty on the start date of the clinical trial trajectory remained, these specific cases were discussed in a meeting with two additional senior investigators (SH, MvdH). During this meeting, a best estimate for the start date was determined, provided there was enough evidence for a date on which the first study in humans must have been done. These alternative start dates (ASD) could be based upon the submission date of an article that published results of the first in human trial or referencing in other articles, a conference during which the first results were presented, the first available phase 1 trial that was not in healthy subjects but in patients; or a phase 2 trial that was performed right after an unpublished first phase 1 trial. Cases without acceptable leads for the start date were excluded from the analysis. When start dates only contained the month and the year, we used the first day of the stated month and year for the start date.

For our secondary analysis, we searched the FDA website for the marketing approval dates of the medicines included in our study, to calculate the median clinical development times for FDA approved medicines.^24^

### Factors associated with clinical development time

Table 1 provides an overview of potential factors contributing to total length of a clinical development period. Factors are placed in overlapping themes which include regulatory and political factors, characteristics of the disease and of the compound tested, trial design and methodology, and finance and organisation. Although this table is simplified as most factors are interrelated or could be placed under multiple themes, it does provide an overview of all the potential factors to be considered, in order to understand the total duration of a clinical trial trajectory when studying systematic acceleration.

**Table 1:** Factors associated with total length of clinical development time from phase 1 to registration.

Theme	Factor
Regulatory and political factors	Laws and regulations of country/continent where trial(s) are performed
	Assessment status granted by regulatory body (EMA/FDA) such as accelerated assessment, CMA, PRIME scheme, orphan drug designation
	Political declaration of emergency outbreak/outbreak setting
	Number of trials performed (or requested) before registration approval by regulatory body
Disease characteristics	Type of disease targeted
	Disease prevalence or incidence (availability of participants)
	Duration of treatment
Compound characteristics	Type of treatment (curative, preventive, suppressive)
	Combination of medical compounds (combination pill), or rebranding, reusing, repurposing
	Large-scale production capabilities
	Testing of generic compound
Trial design/methodology	Ethical approval
	Type of study design (e.g. adaptive trial platform, single-centre)
	Combined or overlapping phases
	Duration of recruitment
	Choice of endpoints (surrogate versus clinical endpoints)
	Duration of observation until the primary endpoint for registration
Funding and organisation	Duration of contract negotiations
	Site organisation (staff turnover, employment conditions, career paths, workload, delegation and management)
	Background of sponsor-investigator (pharmaceutical, academic, governmental)
	Time between subsequent clinical trial phases
	Planning (clear project ideas, realistic deadlines, understanding of trial processes, adaptation to the local context and involvement of site staff in planning)
	Funding availability (per phase or per trajectory)/budget feasibility

A list of factors associated with the total length of a clinical development time described in literature.^3^^,^^11^^,^^12^^,^^14^^,^^20^^,^^21^ Factors are placed in overlapping themes.

For all the included registered compounds, we collected data on factors we hypothesized could be associated with a shorter clinical development time (Table 1); however, most factors could not be included in the analysis due to a lack of data availability. Included factors were whether the clinical trial trajectories were performed in response to an outbreak setting of an EID in the decade of our search (2012–2022), as defined by the WHO.^19^ Added to that, we included which pathogen (bacteria, virus, fungi, or other) was targeted with the treatment, and type of disease(s) it targeted, a distinction was made between vaccines and drugs, if the registered compound contained more than one substance (combination drug), and the sponsor-investigator of the first phase 1 trial (academia, pharmaceutical industry or governmental). Finally, regulatory factors used by the EMA, such as CMA, accelerated assessment, and orphan drug designation were included. Most of these data were available within the EMA EPAR database, with the exception of the outbreak setting which was retrieved from the WHO list of global emergencies.^19^

### Data analysis

The data were analysed using StataSE 16 (Texas, Houston). We described our data using means (standard deviations [SD]), medians (interquartile range [IQR]) and proportions, as appropriate. P-values below 0.05 were considered statistically significant.

Development times of the clinical trial trajectories were calculated in years by subtracting the phase 1 start date from the first marketing approval date and divided by 365.25. We calculated the clinical development times using the definitive start dates and the alternative start dates and compared their distributions. If they were similar, we analysed them as a whole. We stratified clinical development times by disease type.

We performed uni- and multivariable linear regression analysis of the association with clinical development time (in years) to calculate unadjusted and adjusted regression coefficients with 95% confidence intervals (CI), respectively. In the multivariable model we included all a priori hypothesized factors associated with the outcome and which were available to us.

For the sensitivity analysis, we repeated the multivariable model excluding COVID-19 compounds. For the comparison of clinical development times at EMA versus FDA, we calculated the FDA development time in the same manner as the EMA replacing the FDA registration date; and calculated the median time overall and per compound, and compared these using the Wilcoxon ranksum test.

### Role of the funding source

The funder of the study had no role in study design, data collection, data analysis, data interpretation, or writing of the report.

## Results

### Data selection

**Fig. 2:**
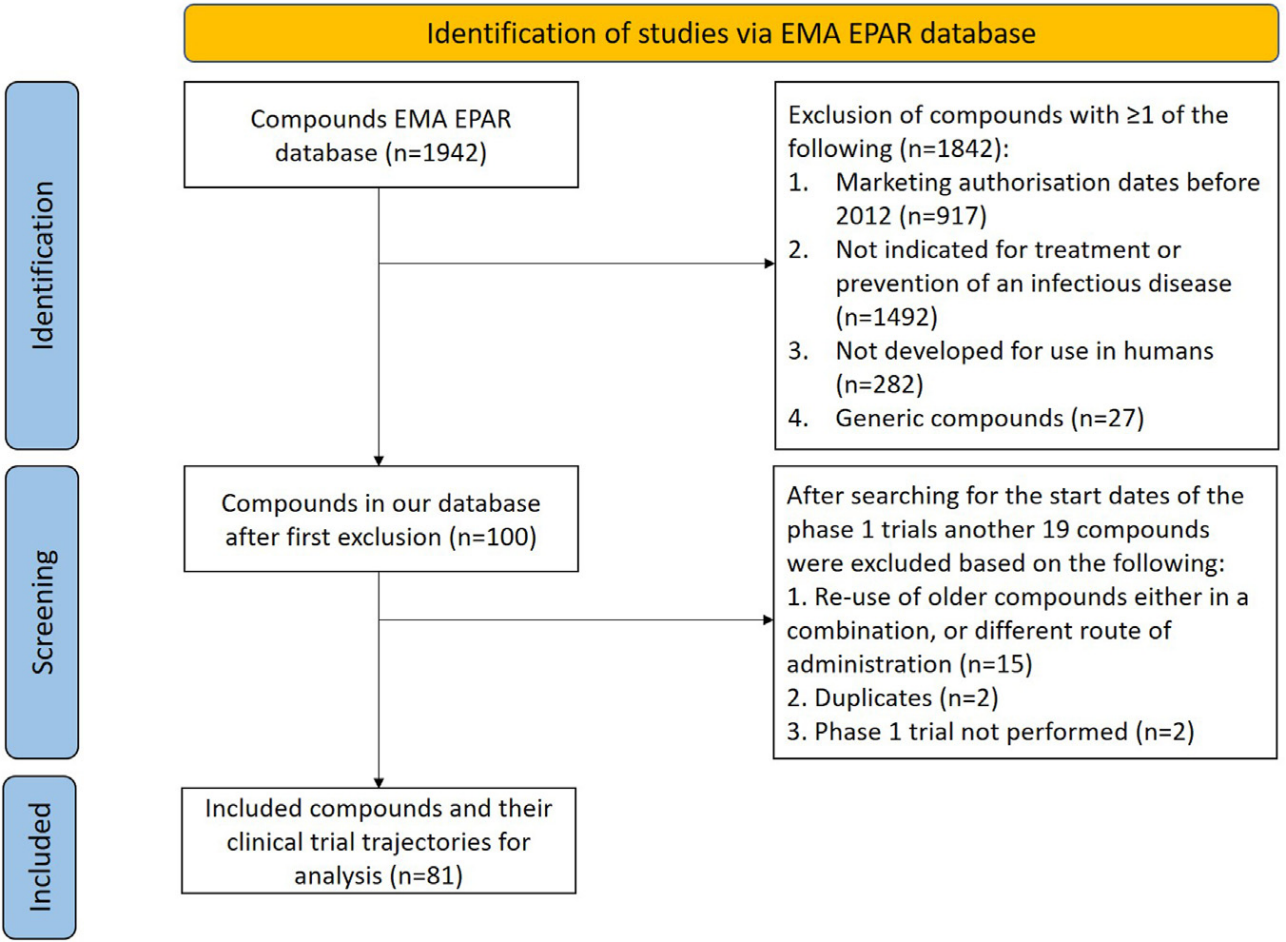
**Flowchart of in- and excluded EMA-registered compounds**. The EMA EPAR database was accessed on 12-09-2022 and contained 1942 pharmacotherapeutical compounds. Compounds were excluded with at least one of the following exclusion criteria: not licensed before the year 2012, were not indicated for treatment of prevention of infectious diseases, were not developed for use in humans, or contained generic medicines. After the first round of exclusion, search for phase 1 start dates was performed via the 3-step process, and another 19 compounds were excluded based on the following criteria: re-use of older compounds either in a combination or different application, duplicates, and phase 1 trials were not performed.

The EMA EPAR database accessed on 12-09-2022 contained 1942 pharmacotherapeutic compounds which were registered between 2012 and 2022. After applying all the exclusion criteria, 100 compounds were checked for the start date of the phase 1 trial via our 3-step process (see Methods section; and Fig. 2 for an overview of the selection process).

Pharmaceutical companies were contacted if it was not possible to collect the start date at the first attempt (n = 44). From the pharmaceutical companies that were contacted, 22/44 (50%) responded; out of which 8/22 (36%) were able to provide useful information. Reasons for not being able to share the data were because they did not keep track of this information; because they did not want to share this information; or because they did not respond to follow up questions. After the search by means of our 3-step process was completed, 59/81 (73%) of compounds had a definitive start date, and 22/81 (27%) an alternative start date. From the 100 compounds included in the initial search, 19 were excluded due to the following reasons: re-use of older registered medicine (rebranding, n = 15), duplicates (n = 2), and phase 1 trial was not performed (n = 2). Thus, in total, 81 compounds were included for analysis.

### Baseline characteristics

Table 2 shows the baseline characteristics for the 81 clinical trial trajectories included. The majority were drugs 53/81 (65%) rather than vaccines; and targeted a virus 53/81 (65%) rather than a bacterial infection. In total 19/81 (24%) of the medicines were combinations of more than one medical compound. Table 2, the frequencies of the observed targeted infectious diseases are depicted, with HIV infections, hepatitis (A to E) and COVID-19 making up the greater part of the viral infectious diseases targeted. Over a quarter of the trajectories were performed under the circumstances of an EID outbreak (23/81; 28%). The majority of the trajectories were initiated and sponsored by a pharmaceutical company (73/81; 90%). Expedited programmes such as CMA, orphan drug designation or accelerated assessment granted by the EMA applied to 11/81 (14%), 8/81 (10%) and 6/81 (7%), respectively.

**Table 2:** Baseline characteristics of the included compounds.

Characteristic	Total (N = 81)
	n (%)
Vaccine	
Yes	28 (34.6)
No	53 (65.4)
Combination drug	
Yes	19 (23.5)
No	62 (76.5)
Pathogen(s) targeted	
Virus	53 (65.4)
Bacteria	25 (30.9)
Fungi, other	3 (3.7)
Outbreak setting	
EID	23 (28.4)
Non-EID	58 (71.6)
Type of sponsor-investigator of phase 1 trial	
Academic	2 (2.5)
Pharmaceutical	73 (90.1)
Governmental	6 (7.4)
CMA	
Yes	11 (13.6)
No	70 (86.4)
Orphan drugs	
Yes	8 (9.9)
No	73 (90.1)
Accelerated assessment	
Yes	6 (7.4)
No	75 (92.6)
Type of disease	
HIV	15 (18.5)
COVID-19	12 (14.8)
Hepatitis (A-E)	12 (14.8)
Influenza	5 (6.2)
DR-TB	3 (3.7)
Ebola virus disease	3 (3.7)
Other bacterial disease^a^	22 (27.2)
Other^b^	9 (11.1)

EID; Emerging infectious disease. CMA; Conditional marketing authorisation. DR-TB; drug-resistant tuberculosis.

a Including vaccines targeting *meningococci*, *pneumococci,* and *Vibrio cholera,* and antibiotics or monoclonal antibodies targeting *Gram-negative* bacterial infections, respiratory tract infections, community acquired bacterial infections, intra-abdominal infections, and anthrax.

b Including vaccines targeting human papilloma virus, a combination of diphtheria/acellular pertussis/tetanus/polio/haemophilus influenzae b/hepatitis B, poxviridae infections, herpes zoster, and dengue, and antiviral targeting cytomegalovirus, poxviridae infections, and antimycotics targeting aspergillosis.

### Clinical development time

Development times were calculated for trajectories with DSD (59/81, 72.8%); median development time 7.7 (IQR 4.5–12.7) years; and ASD (22/81, 27.2%); median development time 7.1 (IQR 4.3–9.7) years. As the medians and distributions did not differ much, it was decided to analyse the data together, assuming the ASD for those without a DSD.

The median clinical development time was 7.3 (IQR 4.4–12.3) years. The fastest trajectories were eight months for the COVID-19 vaccines registered by Janssen-Cilag and BioNTech/Pfizer. The longest trajectory was 223 months for oritavancin (Tenkasi®), an antibiotic for the treatment of skin infections. A prior applicant sought approval for oritavancin in 2008 but this application was withdrawn. The manufacturing process had to be adjusted multiple times and was considered as non-standard. While the initial start date was in 1996, final authorisation was issued at the start of 2015, possibly explaining the extended development time.^25^

**Fig. 3:**
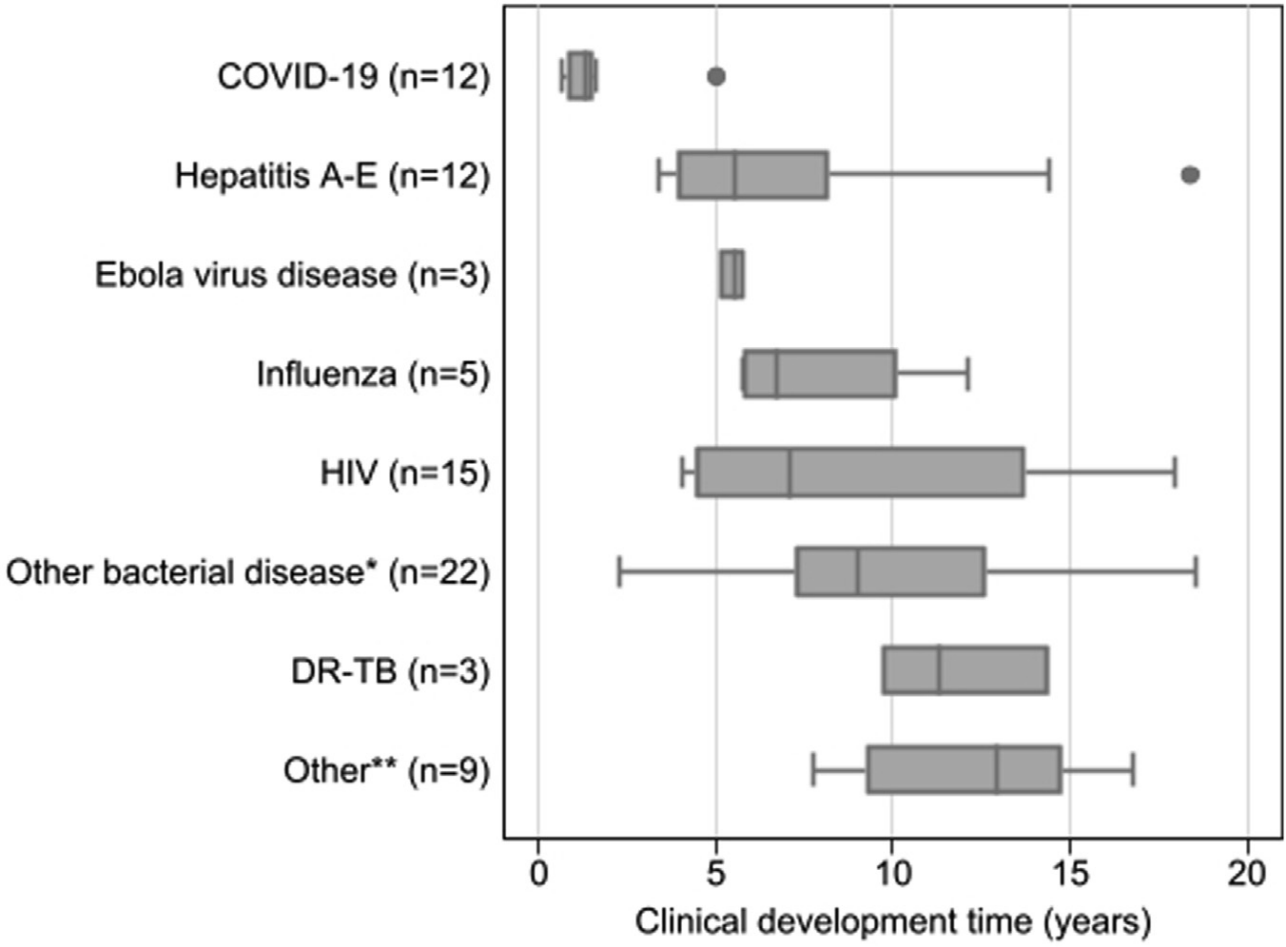
**Clinical development times per disease targeted, in years (median, IQR)**. Box plot figure. Note. IQR, interquartile range; DR-TB, drug-resistant tuberculosis. ∗Including vaccines targeting *meningococci, pneumococci,* and *Vibrio cholerae,* and antibiotics or monoclonal antibodies targeting Gram-negative bacterial infections, respiratory tract infections, community acquired bacterial infections, intra-abdominal infections, and anthrax). **∗∗**Including vaccines targeting human papilloma virus, a combination of diphtheria/acellular pertussis/tetanus/polio/haemophilus influenzae b/hepatitis B, poxviridae infections, herpes zoster, and dengue, and antivirals targeting cytomegalovirus, poxviridae infections, and antimycotics targeting aspergillosis.

Fig. 3 shows the median duration of development time per type of disease. Almost all medicines targeting an infectious disease had a median clinical development time shorter than ten years, with the exception of DR-TB drugs and medicines falling under the category ‘other’ (which includes some vaccines targeting viruses such as dengue and herpes zoster, antifungal medicines, and combination vaccines targeting both viruses and bacteria). Noteworthy are the medicines developed for COVID-19 with a median duration of 1.3 (IQR 0.8–1.6) years, and those for EVD with a median duration of 5.5 (IQR 3.5–8.2) years.

### Accelerated trajectories

Table 3 displays the 20 fastest trajectories; for the full list of all included trajectories see the Supplementary Table S1. The quickest trajectories took only eight months and were all COVID-19 vaccines. Even the top-11 fastest vaccine and drug trajectories were all from the COVID-19 era, with 19 months being the lengthiest of these. However, not only COVID-19 trajectories exhibited faster-than-average clinical development times. These included trajectories for drugs for treatment of HIV (3/20, 15%) or hepatitis C (5/20, 25%), and the prevention of meningitis B by the meningococcal group B Vaccine (rDNA, component, adsorbed (Bexsero®) vaccine (27 months).

**Table 3:** The twenty shortest clinical development times for compounds targeting infectious diseases approved by the European Medicines Agency, 2012–2022.

Compound(s)	Disease targeted	Development time (months)	Start date phase 1 (dd-mm-yyyy)	EMA marketing authorisation date (dd-mm-yyyy)
COVID-19 vaccine Ad26.CoV2–S [recombinant](V)^a^	COVID-19	8	15-07-2020	11-03-2021
Tozinameran/riltozinameran (V)^b^	COVID-19	8	23-04-2020	21-12-2020
COVID-19 vaccine (ChAdOx1 S [recombinant]) (V)^c^)	COVID-19	9	23-04-2020	29-01-2021
COVID-19 mRNA vaccine (nucleoside modified) (V)^d^)	COVID-19	10	16-03-2020	6-01-2021
Nirmatrelvir + ritonavir (D)^e^	COVID-19	12	11-02-2021	28-01-2022
Sotrovimab (D)^f^	COVID-19	16	27-08-2020	17-12-2021
Regdanvimab (D)^g^	COVID-19	16	18-07-2020	12-11-2021
Casirivimab, imdevimab (D)^h^	COVID-19	17	11-06-2020	12-11-2021
COVID-19 vaccine (inactivated, adjuvanted, adsorbed) (V)^i^	COVID-19	18	16-12-2020	24-06-2022
COVID-19 vaccine (SARS-CoV-2 rS [Recombinant, adjuvanted]) (V)^j^	COVID-19	19	25-05-2020	20-12-2021
Tixagevimab, cilgavimab (D)^k^	COVID-19	19	18-08-2020	25-03-2022
Meningococcal group B vaccine (rDNA, component, adsorbed) (V)^l^	Meningitis B	27	21-10-2010	13-01-2013
Sofosbuvir, velpatasvir, voxilaprevi(D)^m^	Hepatitis C	40	13-03-2014	26-07-2017
Sofosbuvir, elpatasvir(D)^n^	Hepatitis C	44	6-11-2012	6-07-2016
Glecaprevir, pibrentasvir(D)^o^	Hepatitis C	45	01-11-2013	26-07-2017
Sofosbuvir(D)^p^	Hepatitis C	48	10-01-2010	16-01-2014
Bictegravir, emtricitabine, tenofovir alafenamide (D)^q^	HIV	48	11-06-2014	21-06-2018
Elvitegravir, cobicistat, emtricitabine, tenofovir disoproxil(D)^r^	HIV	49	01-05-2009	24-05-2013
Lenacapavir(D)^s^	HIV	49	20-7-2018	17-08-2022
Ledispavir, sofosbuvir(D)^t^	Hepatitis C	52	01-08-2010	17-11-2014

Note. List in descending order of clinical trial development times (months). Dates depicted as (dd-mm-yyyy). For the full list, see Appendix Supplemental Table S1. D, drug; V, vaccine; HIV, Human Immunodeficiency Virus.

a Jcovden® (Janssen-Cilag International NV).

b Comirnaty® (BioNTech Manufacturing GmbH).

c Vaxzevria® (AstraZeneca AB).

d Spikevax® (Moderna Biotech Spain, S.L.).

e Paxlovid® (Pfizer Europe MA EEIG).

f Xevudy® (GlaxoSmithKline Trading Services Limited).

g Regkirona® (Celltrion Healthcare Hungary Kft.).

h Ronapreve® (Roche Registration GmbH).

i Valneva® (Valneva Austria GmbH).

j Nuvaxovid® (Novavax CZ, a.s.).

k Evusheld® (AstraZeneca AB).

l Bexsero® (GSK Vaccines S.r.l.).

m Vosevi® (Gilead Sciences Ireland UC).

n Epclusa® (Gilead Sciences Ireland UC).

o Maviret® (AbbVie Deutschland GmbH Co. KG).

p Sovaldi® (Gilead Sciences Ireland UC).

q Biktarvy® (Gilead Sciences Ireland UC).

r Stribild® (Gilead Sciences Ireland UC).

s Sunlenca® (Gilead Sciences Ireland Unlimited Company).

t Harvoni® (Gilead Sciences Ireland UC).

The Ebola vaccines Ebola Zaire vaccine (rVSVΔG-ZEBOV-GP, live) (Ervebo®), Ebola vaccine (Ad26.ZEBOV-GP [recombinant]) (Zabdeno®), and Ebola vaccine (MVA-BN-Filo [recombinant]) (Mvabea®) did not belong to the top-20 fastest trajectories, yet they were faster than the median with development times of 61, 66, and 69 months, respectively.

### Factors associated with clinical development time

Table 4 shows the results of the univariable and multivariable regression analysis. An EID setting was associated with a 5.4-year (95% CI 2.6–8.2 years) reduction in clinical development time compared to a non-EID setting. Also associated with a reduced clinical development time was the development of a combination drug compared to a single drug (2.7 years, 95% CI 0.4–4.9). When looking at specific statuses given by the EMA, both the granting of CMA and accelerated assessment were associated with a shorter development time in the univariable analysis, but only accelerated assessment remained associated in the multivariable analysis with a four-year shorter development time (95% CI 0.5–7.6 years). Orphan drug status was associated with a 3.9 (95% CI 0.4–7.4) lengthening of the clinical development time in the univariable analysis; however, this association was not significant in the multivariable analysis. No other factors were associated with clinical development time length. Excluding COVID-19 compounds in our sensitivity analysis did not lead to a different outcome, the significant time reduction remained for the earlier mentioned factors although the factor EID was associated with a reduced time of 4.1 (95% CI 0.4–7.8) years (Supplementary Table S2).

**Table 4:** Linear regression analysis of factors associated with clinical development time length.

Variable	Univariable	P	Multivariable	P
**Outbreak setting**				
aEID	Ref		Ref	
Non-EID	−4.7 (−6.8 to −2.6)	<0.001	−5.4 (−8.2 to −2.6)	**<0.001**
**Pathogen(s) targeted**				
Virus	Ref		Ref	
Bacteria	3.0 (0.8–5.2)	0.008	0.3 (−1.9 to 2.4)	0.82
Other/mix/fungi	5.3 (0.1–10.7)	0.054	1.2 (−3.8 to 6.3)	0.48
**Vaccine**				
Yes	Ref		Ref	
No	1.5 (−0.7 to 3.7)	0.18	0.2 (−2.1 to 2.5)	0.86
**Combination drug**				
No	Ref		Ref	
Yes	−2.7 (−5.2 to 0.3)	0.03	−2.7 (−4.9 to −0.4)	**0.02**
b**CMA status**				
No	Ref		Ref	
Yes	−3.1 (−6.1 to −0.1)	0.046	−1.0 (−4.4 to 2.3)	0.53
**Orphan drug**				
No	Ref		Ref	
Yes	3.9 (0.4–7.4)	0.02	3.1 (−0.6 to 6.8)	0.10
**Accelerated assessment**				
No	Ref		Ref	
Yes	−3.5 (−7.5 to 0.5)	0.08	−4.0 (−7.6 to −0.5)	**0.03**
**Type of sponsor-investigator of phase 1 trial**				
Pharmaceutical	Ref		Ref	
Academic	−4.9 (−11.8 to 1.9)	0.16	−0.8 (−6.7 to 5.1)	0.79
Governmental	0.6 (−3.5 to 4.7)	0.76	2.5 (−1.3 to 6.2)	0.19

*P*-values below 0.05 were considered statistically significant (bold).

a EID: emerging infectious disease.

b CMA: conditional marketing authorisation. CI: 95% confidence interval; Ref, reference category.

Of the 81 included compounds in our study, only 68 were also approved by the FDA (85%).

When comparing the clinical development time of the included compounds of the EMA versus the FDA of those 68 compounds, we found that the median development time for FDA approved compounds was 7.0 (IQR 4.2–11.9) years, and the EMA was 7.7 (IQR 4.4–12.9) years, which was not significantly different (P = 0.78). COVID-19 and HIV targeting compounds were approved on average a little faster by the FDA than the EMA, but the compounds targeting viral hepatitis were approved faster by the EMA (Supplementary Table S3).

## Discussion

We examined the current average clinical development time of infectious disease-related drugs and vaccines registered by the EMA over the past ten years. Among the 81 clinical trial trajectories included, the shortest development times were recorded for registered COVID-19 vaccine and drug clinical trial trajectories. The second-shortest development times were recorded for registered drugs and vaccines targeting Ebola virus disease, and viral hepatitis; both with a median of 5.5 years. Factors that were associated with accelerated times were clinical development during an outbreak setting (EID), development of combination drugs, and the regulatory factor accelerated assessment, a status granted by the EMA. These beneficial factors for time reduction remained associated when excluding COVID-19-related compounds from the analysis. Finally, the median clinical development time of the included compounds in this study did not differ between EMA and FDA.

Clinical development times and examples of acceleration within the clinical trials landscape have been reported in the literature in the fields of vaccine development and oncology, but mainly as descriptive summaries and estimates.^2^^,^^3^^,^^5^^,^^11^^,^^26^ The median clinical development time of EMA approved drugs and vaccines targeting infectious diseases was 7.3 years, which was substantially faster than the 10–15 years for trajectories in general described in the literature.^2^, ^3^, ^4^, ^5^ Our study is in line with the study performed by Brown et al. (2022), which calculated the clinical development times of successful drug development programmes of FDA-approved drugs in the past decade.^3^ In their study the median development time was 8.5 years which was slightly longer than our median. This difference could be explained by the compounds included: Brown et al. (2022) included all disease types, where we only included drugs targeting infectious diseases. They further show that antiviral drugs had the shortest clinical development times and antibacterial drugs the longest, with all other therapeutic classes in between. Although they do not give an explanation for this disparity, one can argue that development of drugs against most infectious diseases is comparatively simple (exceptions are diseases with long incubation, treatment, and follow up times, such as TB and some parasitic diseases). In many chronic diseases, clinical trials have to cover a much larger time for efficacy and safety reasons. Such trials are often conducted with outpatients, which leads to lower compliance, reduced data quality and higher recruitment challenges or have to make use of wide networks of study centres and clinics offering trial participation. Most importantly, endpoints may be soft or experimental, which can make interpretation more challenging and could lead to increased discussion with regulatory agencies. This at least would explain the success of antivirals compared to other therapeutic classes in their study, but does not apply to the clinical development times for antibacterial compounds (unless they only included TB targeting drugs), which appear to have a similar median time as other therapeutic classes (although with a broad range), for which we do not have a suitable explanation.

It is not surprising that the 11 fastest clinical development times were for the registered COVID-19 drugs and vaccines. Four COVID-19 vaccines had the shortest clinical development times, ranging from 8 to 10 months. The rapid development of the adenovirus vector vaccine and the mRNA vaccines could be attributed to the large body of evidence available resulting from previous, limited outbreaks (in case of the adenovirus vaccine: (Zaire) Ebola, influenza, and Zika viruses; in case of mRNA vaccines SARS and MERS viruses). Both vaccine platforms also rely on the delivery of either DNA or mRNA encoding antigens to induce an immune response against the pathogen, and by simply altering the delivered nucleic acid sequence can lead to the rapid development of a novel vaccine.^27^ However, trajectories of treatments of other infectious diseases were also faster than the average; i.e., for the treatment or prevention of HIV, hepatitis C, and meningitis B. The rapid clinical development times of drugs targeting hepatitis C were not driven by an outbreak situation but because of a scientific breakthrough; the development of NS5A inhibitors in 2010. This entirely new drug class was considered a major milestone towards HCV cure, and led to the development of interferon-free therapies. Multiple pharmaceutical companies quickly designed their own direct-acting antiviral (DAA) regimen containing a NS5A inhibitor and applied for accelerated assessment with the EMA. Initial approval based on a pre-selection of patient groups ineligible for interferon treatment, following high clinical success rates and little safety issues, also contributed to the accelerated approvals of DAA's.^28^

The outbreak of an EID had the biggest impact on the clinical development time which is in line with literature on acceleration of drug development during the COVID-19 pandemic.^29^^,^^30^ There are several potential explanations for why an outbreak enables rapid development of medicines. Once an EID is declared a global public health emergency, a heightened sense of urgency is created. The search for a preventive or curative treatment strategy will be hastened due to a disease's quick spread and high morbidity or mortality. This leads to both funding and resources to receive priority, allowing for more research to be carried out, and more trials to be initiated.^29^ In the light of the COVID-19 pandemic, considerable social and economic impact led to governments providing sizable funding for vaccine research, enabling researchers to pursue the rapid development of vaccine candidates (ultimately leading to the first mRNA vaccines) in order to contain the disease.^31^ Some may argue that only with similar funding and manpower available will it be possible to replicate the rapid achievement during an outbreak, and that this will only be forthcoming if a similar sense of social and political urgency exists.^29^ This seems unlikely to be feasible in a non-outbreak setting. For example, the Coalition for Epidemic Preparedness Innovations (CEPI), a global partnership initiated after the poor global response to the 2014–2016 West Africa Ebola epidemic, working to accelerate vaccine development, tries to maintain this sense of urgency created during that time. They have successfully raised money to continuing working on pandemic preparedness by strengthening global alignment (including organizing trial networks, global surveillance, and further data collaboration such as sharing libraries of vaccine constructs).^11^ However, the goals of CEPI will only be met with sufficient funding and governmental empowerment; and will therefore be only useful for the development of drugs and vaccines targeting an epidemic or pandemic threat for which enough political urgency exists.

Another associated regulatory and political factor found in our study was being granted accelerated assessment by the EMA, a mechanism offered to expedite reviews on applications for marketing authorisation which is comparable to the Priority Review program of the FDA.^32^^,^^33^ It usually takes two years or longer for a drug to reach the patient from the moment of application for authorisation by the EMA. Although the EMA application process allows for a maximum of 210 days turnaround time per product; after authorisation, there are still several steps required before the product can be prescribed to a patient.^34^ This shows that progress can be made when streamlining the regulatory pathway by granting products a special status; therefore, at least shortening the EMA reviewing time. Accelerated assessment was introduced by the revised EU pharmaceutical legislation in November 2005. The aim of this regulatory tool was to help speed up access to new medicines of major public-health interest by a faster and more efficient review. Interestingly, accelerated assessment was not used for the clinical trials of drugs targeting an EID in our analysis, although these could also be defined as of public health interest.

Another approach by the EMA that tries to ensure early access to new medicines is CMA, for which medicines are eligible if they are intended for treating, preventing or diagnosing seriously debilitating or life-threatening diseases, and which could also be used for a public health emergency. Such medicines receive CMA based on preliminary and less comprehensive data, provided that clinical trials continue, and additional data are generated after this conditional authorisation.^35^ ‘Rolling reviews’ as part of the CMA scheme were used throughout the COVID-19 pandemic, which involved assessing clinical data from ongoing trials as soon as they became available.^36^ Many SARS-CoV-2 targeting drugs and vaccines were given this CMA status, which ultimately led to accelerated trajectories; although not all of the accelerated COVID-19 targeting medicine from our top-20 had a CMA status. We found no association between CMA status granted and a reduction in clinical development time over the past decade, which perhaps has to do with the fact that there were multiple schemes for expedited reviews, such as accelerated assessment described above. Interestingly, CMA status was being granted for drugs targeting an EID in our analysis, instead of the earlier-mentioned accelerated assessment status; although both could be used when reading the criteria and conditions.

Orphan drug status granted for a medicine that targets rare medical conditions, face more challenges during the clinical development process compared to traditional medicines (smaller patient population size, complex study design, limited understanding of the exact pathophysiology) and renders them therefore less attractive to develop. Although the designation of an ‘orphan drug’ status cannot mitigate these difficulties, the sponsor is being offered incentives by the EU (e.g., being granted CMA; receiving ten-year market protection) to start development despite these difficulties. Although the prolonged clinical trial timeline remains a persistent challenge in literature, we did not find an association between orphan designation and a lengthening on the clinical development in our analysis.^3^

Last, both accelerated assessment and CMA status are granted separately, or can be part of the PRIME scheme, where priority medicines that target an ‘unmet medical need’ receive tailor-made advice, guidance, and evaluation to enhance speedy development. We were unable to analyse the impact of the PRIME scheme as the EMA launched this scheme only in 2016, and none of our included trajectories made use of this scheme. Access to any of these priority statuses is only granted if the medicine is considered to be of ‘major public health interest’ or targeting an ‘unmet medical need’.^37^, ^38^, ^39^ However, this can be interpreted liberally as the EMA has not established clear criteria on these definitions.^40^ Furthermore, 71% percent of PRIME requests are rejected.^37^ This begs the question of how equitable the allocations of these statuses are; and whether not more medicines should be granted such priority statuses in the future.

Similar to the PRIME scheme are the FDA's Breakthrough Therapy Designation and Fast Track designation, regulatory tools that have existed for more than a decade and have contributed significantly to expedited drug development.^3^^,^^41^ When comparing the registration dates between the FDA and the EMA, there was little difference in clinical development time for medicines targeting infectious diseases. In our analysis we were not able to compare the different expedited pathways of the regulatory bodies used per compound. However, in a recent report by the Centre for Innovation and Regulatory Science (CIRS), a comparison between six major regulatory agencies showed that in 2021 the ratio of expedited approvals to standard reviews was highest with 71% for the FDA, but considerably lower for the EMA (9%).^42^ An explanation given is that medicines, when assigned an expedited program by the EMA, can still revert to standard review when targets are not met, which is not possible for FDA fast-tracks. However, expedited pathways, especially combining different programs, have shown to generate the largest impact on both development and review timelines, and leads to competitive advantages between regulatory agencies.^41^^,^^42^ The influence of the development of regulations imposed by the EMA on the clinical landscape is substantial. In January 2022, the ACT EU workplan was launched to set out deliverables and timelines for the upcoming years aiding in the transformation of the EU clinical trial landscape, to further promote the development of high quality, safe and effective medicines, and to better integrate clinical research in the European health system. This is currently being studied by our group (manuscript in development), and is therefore not further elaborated on here in the discussion.

The development of combination drugs (these could contain compounds containing one or two already registered drugs with a novel compound) was also associated with a reduced development time. A possible explanation is that the combination drug is in some respects already partly established because a new chemical entity is added to an already authorised compound. This could jump-start the development of a new drug, and reduce the number of trials needed for each phase; as earlier studies can be used to support the evidence for the marketing authorisation application. Most often, experience has already been obtained with similar patient groups when testing the previous compound as mono-treatment, or when tested with other similar compounds. For example, in the case of the accelerated hepatitis C drug trajectories, the discovery of the DAA sofosbuvir led to multiple combination drugs to be developed with this compound, all around the same time period.^28^

There are several limitations to the findings presented in this study. First, a relatively small sample size of 81 clinical trial trajectories remained after exclusion, leading to low power to detect associations. Moreover, nearly a quarter had an alternative start date that was based on a best estimate which may have led to a biased estimate of development times. However, due to comparable medians and distributions, the effect of this is expected to be small and unlikely to affect the results. Secondly, only a limited set of possible factors associated with the length of clinical development times were available for analysis. This was in part due to the difficulty in obtaining sufficient and complete information on clinical trials, despite public databases such as ClinicalTrials.gov, as well as to the low response rate that was encountered when seeking direct contact with pharmaceutical companies asking them for this information. For instance, data on the number of trials of each phase that were conducted during a trajectory, or the time it took for trials to move from phase 2 to phase 3 were very difficult to acquire, and could give a more detailed perspective on clinical development time. Additionally, decisions taken, and resources being made available throughout clinical development unquestionably depend on financial and (geo-) political factors but are challenging to pinpoint for analysis. As a result, we were not able to include some of the a priori identified factors that could be associated.

Furthermore, we only included drugs and vaccines receiving marketing approval by the EMA, using their EPAR database, and the findings are thus limited to the European market. It was chosen to limit the trajectories to vaccines and drugs that target infectious disease. This means our findings are only generalizable to the drugs and vaccines in the therapeutic field of infectious diseases. However, even focussing solely on treatments targeting infectious diseases it is difficult to compare development times, as the relevant study endpoints and treatment and follow up times vary. During the timeframe of our analysis only three products targeting parasites were reviewed by the EMA via a different established review process (EU-M4all) by the EMA in collaboration with the WHO, to provide a positive endorsement of interventions to be licensed in low- and middle-income countries, and could therefore not be included in our analysis.^43^

Lastly, while the past ten years is a relatively recent time frame, it may not accurately reflect long-term trends in acceleration of clinical trial trajectories and its potential drivers. The clinical trial landscape is constantly evolving, and the factors influencing the clinical development time may have altered over time. Hence, it is important the bear in mind the limited time frame and the possibility of changing trends. Drug and vaccine development is a complex and multifactorial process where numerous factors continue to influence the clinical development time.

What could be learned from the current clinical trial development process and what would it take to sustainably accelerate clinical development for drugs and vaccines targeting infectious diseases? Abovementioned factors that were associated with reduced clinical development times are generalizable or modifiable to a limited extent only; and are therefore of limited use to accelerate clinical trials for all drugs and vaccines. In order to identify modifiable factors, it would be necessary to conduct a more extensive analysis of the clinical trial trajectories, which would entail additional data collection on all factors displayed in Table 1. Particularly factors in the overlapping themes trial design and methodology, and finance and organisation, were underrepresented due to a lack of publicly available data. Acceleration of specific parts of the trajectory would be modifiable on individual trial sponsor/trial site level (e.g., time between different-phase trials, timeline of contract negotiations, etc.), but could not be investigated due to lack of data availability. It would also be wishful to try to disentangle common processes underlying the factors in Table 1; for example, a WHO-declared emerging outbreak could lead to increased funding mobilisation. Also, an adverse effect of acceleration of some drugs or vaccines could cause deceleration of others, due to the increased priority and therefore shift of workforce, administration capacity, and funding. Finally, additional research on factors slowing down the developmental process (e.g., orphan drugs) may shine a light on which obstacles or barriers are in the way of acceleration. It is essential that stakeholders are involved in this research to provide data transparency, and to streamline the regulatory pathways before sustainable acceleration could be made feasible for all drugs and vaccines.

### Conclusion

The median clinical development time for EMA registered drugs and vaccines targeting infectious diseases over the past ten years was shorter than previously reported in the literature. Identified factors associated with shorter development times included the outbreak of an EID, streamlining the regulatory pathway by an accelerated assessment status, and trialling combination drugs, and remained significant when removing COVID-19 compounds from the analysis. However, facilitating development time reduction for all clinical trial trajectories requires a more extensive analysis in search of common and modifiable factors.

## Contributors

HKdJ, SMH, and MPG conceived the study. MPG is the guarantor of this work. HKdJ, SMS, and MO extracted the data. HKdJ, SMS, SMH, and MO conducted the primary analysis. HKdJ, SMS, and SMH wrote the first draft of the manuscript and MO, MAvdH, and MPG reviewed subsequent versions. All authors contributed to the conception and design of the study. All authors contributed to data interpretation and analysis. All authors had full access to all the data in the study, contributed to the final version of the manuscript, and had final responsibility for the decision to submit for publication.

## Data sharing statement

Post-publication, the study data, not already submitted as supplementary file, will be made available to researchers upon request (email: h.k.dejong@amsterdamumc.nl).

## Declaration of interests

None declared.

## References

[1] Basics about clinical trials. https://www.fda.gov/patients/clinical-trials-what-patients-need-know/basics-about-clinical-trials.

[2] Thanh Le T., Andreadakis Z., Kumar A. (2020). The COVID-19 vaccine development landscape. Nat Rev Drug Discov.

[3] Brown D.G., Wobst H.J., Kapoor A., Kenna L.A., Southall N. (2022). Clinical development times for innovative drugs. Nat Rev Drug Discov.

[4] Han S. (2015). Clinical vaccine development. Clin Exp Vaccine Res.

[5] Martin L., Hutchens M., Hawkins C. (2017). Trial watch: clinical trial cycle times continue to increase despite industry efforts. Nat Rev Drug Discov.

[6] The drug development process. https://www.fda.gov/patients/learn-about-drug-and-device-approvals/drug-development-process.

[7] Noor N.M., Love S.B., Isaacs T., Kaplan R., Parmar M.K.B., Sydes M.R. (2022). Uptake of the multi-arm multi-stage (MAMS) adaptive platform approach: a trial-registry review of late-phase randomised clinical trials. BMJ Open.

[8] Torok M.E., Underwood B.R., Toshner M. (2021). Challenges and opportunities for conducting a vaccine trial during the COVID-19 pandemic in the United Kingdom. Clin Trials.

[9] Al M., Levison S., Berdel W.E., Andersen D.Z., Decentralised Clinical Trials Task F (2023). Decentralised elements in clinical trials: recommendations from the European Medicines Regulatory Network. Lancet.

[10] Singh A., Khillan R., Mishra Y., Khurana S. (2022). The safety profile of COVID-19 vaccinations in the United States. Am J Infect Control.

[11] Saville M., Cramer J.P., Downham M. (2022). Delivering pandemic vaccines in 100 Days - what will it take?. N Engl J Med.

[12] Deming M.E., Michael N.L., Robb M., Cohen M.S., Neuzil K.M. (2020). Accelerating development of SARS-CoV-2 vaccines - the role for controlled human infection models. N Engl J Med.

[13] (2019). The complex journey of a vaccine – the steps behind developing a new vaccine: the international federation of pharmaceutical manufacturers and associations.

[14] Excler J.L., Saville M., Privor-Dumm L. (2023). Factors, enablers and challenges for COVID-19 vaccine development. BMJ Glob Health.

[15] Bache B.E., Grobusch M.P., Agnandji S.T. (2020). Safety, immunogenicity and risk-benefit analysis of rVSV-DeltaG-ZEBOV-GP (V920) Ebola vaccine in Phase I-III clinical trials across regions. Future Microbiol.

[16] Diacon A.H., Pym A., Grobusch M.P. (2014). Multidrug-resistant tuberculosis and culture conversion with bedaquiline. N Engl J Med.

[17] Liu Y., Matsumoto M., Ishida H. (2018). Delamanid: from discovery to its use for pulmonary multidrug-resistant tuberculosis (MDR-TB). Tuberculosis.

[18] Weng H.B., Chen H.X., Wang M.W. (2018). Innovation in neglected tropical disease drug discovery and development. Infect Dis Poverty.

[19] A brief guide to emerging infectious diseases and zoonoses: World Health Organization (2014).

[20] Fogel D.B. (2018). Factors associated with clinical trials that fail and opportunities for improving the likelihood of success: a review. Contemp Clin Trials Commun.

[21] Vischer N., Pfeiffer C., Limacher M., Burri C. (2017). "You can save time if..."-A qualitative study on internal factors slowing down clinical trials in Sub-Saharan Africa. PLoS One.

[22] World Medical Association (2013). World Medical Association Declaration of Helsinki: ethical principles for medical research involving human subjects. JAMA.

[23] European public assessment reports: background and context. https://www.ema.europa.eu/en/medicines/what-we-publish-when/european-public-assessment-reports-background-context.

[24] FDA website. https://www.fda.gov/.

[25] Tenkasi (previously Orbactiv). 08-06-2023. https://www.ema.europa.eu/en/medicines/human/EPAR/tenkasi-previously-orbactiv.

[26] Wong C.H., Siah K.W., Lo A.W. (2019). Estimation of clinical trial success rates and related parameters. Biostatistics.

[27] Mendonca S.A., Lorincz R., Boucher P., Curiel D.T. (2021). Adenoviral vector vaccine platforms in the SARS-CoV-2 pandemic. NPJ Vaccines.

[28] Manns M.P., Maasoumy B. (2022). Breakthroughs in hepatitis C research: from discovery to cure. Nat Rev Gastroenterol Hepatol.

[29] Ball P. (2021). The lightning-fast quest for COVID vaccines - and what it means for other diseases. Nature.

[30] Kashte S., Gulbake A., El-Amin Iii S.F., Gupta A. (2021). COVID-19 vaccines: rapid development, implications, challenges and future prospects. Hum Cell.

[31] Rudolph A., Mitchell J., Barrett J. (2022). Global safety monitoring of COVID-19 vaccines: how pharmacovigilance rose to the challenge. Ther Adv Drug Saf.

[32] Accelerated assessment. https://www.ema.europa.eu/en/human-regulatory/marketing-authorisation/accelerated-assessment.

[33] Hwang T.J., Ross J.S., Vokinger K.N., Kesselheim A.S. (2020). Association between FDA and EMA expedited approval programs and therapeutic value of new medicines: retrospective cohort study. BMJ.

[34] Antonanzas F., Juarez-Castello C.A., Rodriguez-Ibeas R. (2018). EMA Priority Medicines scheme (PRIME): will more paying-for-performance agreements be needed due to immature data?. Eur J Health Econ.

[35] Conditional marketing authorisation. https://www.ema.europa.eu/en/human-regulatory-overview/marketing-authorisation/conditional-marketing-authorisation.

[36] (2022). EMA initiatives for acceleration of development support and evaluation procedures for COVID-19 treatments and vaccines.

[37] (2021). Recommendations on eligibility to PRIME scheme.

[38] (2017). Conditional marketing authorisation: report on ten years of experience at the European Medicines Agency.

[39] (2016). Guideline on the scientific application and the practical arrangements necessary to implement the procedure for accelerated assessment pursuant to Article 14(9) of Regulation (EC) No 726/2004.

[40] (2017). Unmet medical need; definitions and need for clarity.

[41] Franco P., Jain R., Rosenkrands-Lange E., Hey C., Koban M.U. (2023). Regulatory pathways supporting expedited drug development and approval in ICH member countries. Ther Innov Regul Sci.

[42] (2022).

[43] Cavaller B.M., Harvey Allchurch M., Lagalice C., Saint-Raymond A. (2020). The European Medicines Agency facilitates access to medicines in low- and middle-income countries. Expert Rev Clin Pharmacol.

