# Supplementary material for: Factors associated with acceleration of clinical development for infectious diseases: a cross-sectional analysis of 10-year EMA registration data
Source: Lancet Reg Health Eur. 2024 Jun 24;43:100983. doi: 10.1016/j.lanepe.2024.100983 (PMC11255360; doi:10.1016/j.lanepe.2024.100983)
Supplement: Supplementary Tables [file mmc1.docx]

**Factors associated with acceleration of clinical development for infectious diseases: a cross-sectional analysis of 10-year EMA registration data**

*Supplementary Materials*

Hanna K. de Jong^1*^, Sabine M. Hermans^1,2^, Sophie M. Schuitenmaker^1^, Maya Oli^1^, Mariëtte A. van den Hoven^3^, Martin P. Grobusch^1,4,5,6,7^

^1^Amsterdam UMC, location University of Amsterdam, Center for Tropical Medicine and Travel Medicine, Department of Infectious Diseases, Amsterdam Public Health – Global Health, and Amsterdam Institute for Immunology and Infectious Diseases, Amsterdam, The Netherlands

^2^Amsterdam UMC, location University of Amsterdam, Department of Global Health, Amsterdam Institute for Global Health and Development, Amsterdam, The Netherlands

^3^Amsterdam UMC, location VU University Amsterdam, Department of Ethics, Law and Humanities, Amsterdam, The Netherlands

^4^Institute of Tropical Medicine, German Centre for Infection Research (DZIF), University of Tübingen, Tübingen, Germany

^5^Centre de Recherches Médicales en Lambaréné (CERMEL), Lambaréné, Gabon

^6^Masanga Medical Research Unit (MMRU), Masanga, Sierra Leone

^7^Institute of Infectious Diseases and Molecular Medicine (IDM), University of Cape Town, Cape Town, South Africa

Contents

[Supplementary Tables 3](#_Toc168406408)

[Supplementary Table 1. The 81 trajectories by international non-proprietary name, in descending order of clinical development time 3](#_Toc168406409)

[Table S2. Multivariable model excluding COVID-19 compounds 8](#_Toc168406410)

[Table S3. Median clinical development times for FDA approved compounds per disease targeted, in years (median, IQR) compared to EMA 9](#_Toc168406411)

[Table S4: STROBE check-list 10](#_Toc168406412)

# Supplementary Tables

## Supplementary Table 1. The 81 trajectories by international non-proprietary name, in descending order of clinical development time

|  | | | | |
| --- | --- | --- | --- | --- |
|  | | | | |
| **International non-proprietary name (INN)** | **Disease targeted** | **Development time (months)** | **Start date (dd-mm-yyyy)** | **EMA marketing authorisation date (dd-mm-yyyy)** |
| COVID-19 Vaccine Ad26.CoV2-S [recombinant](V)^1^ | COVID-19 | 8 | 15-07-2020 | 11-03-2021 |
| tozinameran/riltozinameran (V)^2^ | COVID-19 | 8 | 23-04-2020 | 21-12-2020 |
| COVID-19 vaccine (ChAdOx1 S [recombinant]) (V)^3^) | COVID-19 | 9 | 23-04-2020 | 29-01-2021 |
| COVID-19 mRNA vaccine (nucleoside modified)(V)^4^ | COVID-19 | 10 | 16-03-2020 | 06-01-2021 |
| nirmatrelvir + ritonavir(D)^5^ | COVID-19 | 12 | 11-02-2021 | 28-01-2022 |
| sotrovimab (D)^6^ | COVID-19 | 16 | 27-08-2020 | 17-12-2021 |
| regdanvimab (D)^7^ | COVID-19 | 16 | 18-07-2020 | 12-11-2021 |
| casirivimab, imdevimab (D)^8^ | COVID-19 | 17 | 11-06-2020 | 12-11-2021 |
| COVID-19 vaccine (inactivated, adjuvanted, adsorbed)(V)^9^ | COVID-19 | 18 | 16-12-2020 | 24-06-2022 |
| COVID-19 vaccine (SARS-CoV-2 rS [Recombinant, adjuvanted])(V)^10^ | COVID-19 | 19 | 25-05-2020 | 20-12-2021 |
| tixagevimab, cilgavimab (D)^11^ | COVID-19 | 19 | 18-08-2020 | 25-03-2022 |
| meningococcal group B Vaccine (rDNA, component, adsorbed)(V)^12^ | Meningitis B | 27 | 21-10-2010 | 13-01-2013 |
| sofosbuvir, velpatasvir, voxilaprevi(D)^13^ | Hepatitis C | 40 | 13-03-2014 | 26-07-2017 |
| sofosbuvir, elpatasvir(D)^14^ | Hepatitis C | 44 | 06-11-2012 | 06-07-2016 |
| glecaprevir, pibrentasvir(D)^15^ | Hepatitis C | 45 | 01-11-2013 | 26-07-2017 |
| sofosbuvir(D)^16^ | Hepatitis C | 48 | 10-01-2010 | 16-01-2014 |
| bictegravir, emtricitabine, tenofovir alafenamide (D)^17^ | HIV | 48 | 11-06-2014 | 21-06-2018 |
| elvitegravir, cobicistat, emtricitabine, tenofovir disoproxil(D)^18^ | HIV | 49 | 01-05-2009 | 24-05-2013 |
| lenacapavir(D)^19^ | HIV | 49 | 20-07-2018 | 17-08-2022 |
| ledispavir, sofosbuvir(D)^20^ | Hepatitis C | 52 | 01-08-2010 | 17-11-2014 |
| cobicistat (D)^21^ | HIV | 53 | 01-05-2009 | 19-09-2013 |
| ombitasvir, paritaprevir, ritonavir (D)^22^ | Hepatitis C | 53 | 01-08-2010 | 14-01-2015 |
| remdesivir(D)^23^ | COVID-19 | 60 | 01-07-2015 | 03-07-2020 |
| Zaire Ebola virus vaccine (rVSV∆G-ZEBOV-GP, live)  (V)^24^ | Ebola Virus Disease | 61 | 13-10-2014 | 11-11-2019 |
| pneumococcal polysaccharide conjugate vaccine (20-valent, adsorbed)(V)^25^ | Pneumococcal Infections | 63 | 03-11-2016 | 14-02-2022 |
| ceftazidime, avibactam(D)^26^ | CA Infections | 65 | 01-02-2011 | 23-06-2016 |
| ebola vaccine (Ad26.ZEBOV-GP [recombinant])(V)^27^ | Ebola Virus Disease | 67 | 17-12-2014 | 01-07-2020 |
| Prepandemic influenza vaccine (H5N1) (whole virion, inactivated,prepared in cell culture)(V)^28^ | Influenza | 69 | 01-06-2006 | 17-02-2012 |
| baloxavir marboxil(D)^29^ | Influenza | 69 | 07-04-2015 | 07-01-2021 |
| Zaire Ebola virus vaccine (MVA-BN-Filo [recombinant])(V)^30^ | Ebola Virus Disease | 69 | 17-09-2014 | 01-07-2020 |
| meropenem, vaborbactam(D)^31^ | Intra-abdominal infections | 72 | 01-12-2012 | 20-11-2018 |
| ceftolozane, tazobactam(D)^32^ | CA Infections | 73 | 01-09-2009 | 18-09-2015 |
| dolutegravir(D)^33^ | HIV | 75 | 01-11-2007 | 16-01-2014 |
| Dasabuvir(D)^34^ | Hepatitis C | 80 | 01-06-2008 | 14-01-2015 |
| quadrivalent influenza vaccine (recombinant, prepared in cell culture)(V)^35^ | Influenza | 81 | 01-03-2014 | 16-11-2020 |
| daclatasvir(D)^36^ | Hepatitis C | 82 | 01-11-2007 | 22-08-2014 |
| abacavir sulfate / dolutegravir sodium / lamivudine(D)^37^ | HIV | 82 | 01-11-2007 | 31-08-2014 |
| doravirine (D)^38^ | HIV | 85 | 21-10-2011 | 22-11-2018 |
| doravirine, lamivudine, tenofovir disoproxil(D)^39^ | HIV | 85 | 21-10-2011 | 22-11-2018 |
| tedizolid phosphate (D)^40^ | CA Infections | 87 | 06-01-2008 | 23-03-2015 |
| simeprevir(D)^41^ | Hepatitis C | 88 | 23-01-2007 | 14-05-2014 |
| human papillomavirus 9-valent vaccine (recombinant, adsorbed)(V)^42^ | Human papilloma virus | 93 | 24-09-2007 | 10-06-2015 |
| elvitegravir(D)^43^ | HIV | 93 | 01-02-2006 | 13-11-2013 |
| letermovir(D)^44^ | Cytomegalovirus | 94 | 01-03-2010 | 08-01-2018 |
| cholera vaccine, oral, live(V)^45^ | Cholera | 96 | 01-04-2012 | 01-04-2020 |
| cefiderocol(D)^46^ | GNB Infections | 98 | 01-03-2012 | 23-04-2020 |
| ceftaroline fosamil(D)^47^ | CA Infections | 99 | 12-05-2004 | 22-08-2012 |
| meningococcal groups A, C, W-135 and Y conjugate vaccine(V)^48^ | Meningitis A,C,W,Y | 104 | 01-09-2003 | 20-04-2012 |
| tobramycin(D)^49^ | Respiratory tract infections | 108 | 24-03-2006 | 18-03-2015 |
| eravacycline(D)^50^ | Intra-abdominal infections | 108 | 21-09-2009 | 20-09-2018 |
| bulevirtide(D)^51^ | Hepatitis D | 109 | 01-07-2011 | 31-07-2020 |
| diphtheria, tetanus, pertussis (acellular, component), hepatitis B (rDNA), poliomyelitis (inactivated) and Haemophilus influenzae type b conjugate vaccine (adsorbed)(V)^52^ | HBV/D/P/P/T/HI | 111 | 19-01-2004 | 17-04-2013 |
| bedaquiline(D)^53^ | DR-TBC | 116 | 01-07-2004 | 05-03-2014 |
| pandemic influenza vaccine (H5N1) (whole virion, inactivated, prepared in cell culture)(V)^54^ | Influenza | 122 | 01-04-2006 | 20-05-2016 |
| imipenem, cilastatin, relebactam(D)^55^ | GNB Infections | 122 | 01-12-2009 | 13-02-2020 |
| lefamulin(D)^56^ | Respiratory Tract Infections | 133 | 01-07-2009 | 27-07-2020 |
| meningococcal group b vaccine (recombinant, adsorbed)(V)^57^ | Meningitis | 135 | 01-03-2006 | 24-05-2017 |
| delamanid(D)^58^ | DR-TBC | 136 | 01-01-2003 | 27-04-2014 |
| peramivir(D)^59^ | Influenza | 146 | 23-02-2006 | 13-04-2018 |
| pneumococcal polysaccharide conjugate vaccine (adsorbed)(V)^60^ | Pneumococcal Infections | 147 | 25-09-2009 | 13-12-2021 |
| smallpox and monkeypox vaccine (Live Modified Vaccinia Virus Ankara)(V)^61^ | Poxviridae Infections | 148 | 01-04-2001 | 31-07-2013 |
| meningococcal group A, C, W-135 and Y conjugate vaccine(V)^62^ | Meningitis A,C,W,Y | 152 | 01-04-2008 | 18-11-2020 |
| delafloxacin(D)^63^ | CA Infections | 152 | 19-04-2007 | 16-12-2019 |
| cabotegravir(D)^64^ | HIV | 155 | 01-02-2008 | 17-12-2020 |
| amikacine(D)^65^ | Respiratory Tract Infections | 155 | 25-11-2007 | 27-10-2020 |
| isavuconazole(D)^66^ | Aspergillosis | 155 | 04-11-2002 | 15-10-2015 |
| fostemsavir(D)^67^ | HIV | 159 | 01-11-2007 | 04-02-2021 |
| herpes zoster vaccine (recombinant, adjuvanted)(V)^68^ | Herpes Zoster | 159 | 14-12-2004 | 21-03-2018 |
| elvitegravir, cobicistat, emtricitabine, tenofovir alafenamide(D)^69^ | HIV | 165 | 01-03-2002 | 19-11-2015 |
| emtricitabine, rilpivirine, tenofovir alafenamide(D)^70^ | HIV | 165 | 30-09-2002 | 21-06-2016 |
| elbasvir, grazoprevir(D)^71^ | Hepatitis C | 173 | 01-03-2002 | 22-07-2016 |
| pretomanid(D)^72^ | DR-TBC | 173 | 01-03-2006 | 31-07-2020 |
| diphtheria, tetanus, pertussis (acellular, component), hepatitis B (rDNA), poliomyelitis (inactivated) and Haemophilus type b conjugate vaccine (adsorbed)(V)^73^ | HBV/D/P/P/T/HI | 178 | 01-05-2001 | 15-02-2016 |
| tenofovir alafenamide(D)^74^ | HIV | 178 | 01-03-2002 | 09-01-2017 |
| tecovirimat monohydrate(D)^75^ | Poxviridae Infections | 179 | 01-02-2007 | 06-01-2022 |
| obiltoxaximab(D)^76^ | Anthrax | 180 | 14-11-2005 | 18-11-2020 |
| dalbavancin(D)^77^ | CA Infections | 187 | 01-08-1999 | 19-02-2015 |
| dengue tetravalent vaccine (live, attenuated)(V)^78^ | Arbovirus (Dengue) | 201 | 05-03-2002 | 12-12-2018 |
| ibalizumab(D)^79^ | HIV | 216 | 01-10-2001 | 26-09-2019 |
| hepatitis B surface antigen(V)^80^ | Hepatitis B | 221 | 02-10-2002 | 18-02-2021 |
| oritavancin(D)^81^ | CA Infections | 223 | 01-9-1996 | 18-03-2015 |

Note. List in descending order of clinical trial development times (months). Dates depicted as (dd-mm-yyyy). D, drug; V, vaccine; HIV, Human Immunodeficiency Virus; CA-infections, community-acquired infections; GNB-infections, Gram-negative bacterial infections; HBV/D/P/P/T/HI, hepatitis B/diphteria/polio/pertussis/tetanus/*Haemophilus influenzae*; DR-TB, drug-resistant tuberculosis.

List in descending order of cycle times (months). Dates depicted as (dd-mm-yyyy).

^1^Jcovden® (Janssen-Cilag International NV)

^2^Comirnaty® (BioNTech Manufacturing GmbH)

^3^Vaxzevria® (AstraZeneca AB)

^4^Spikevax® (Moderna Biotech Spain, S.L.)

^5^Paxlovid® (Pfizer Europe MA EEIG)

^6^Xevudy® (GlaxoSmithKline Trading Services Limited)

^7^Regkirona® (Celltrion Healthcare Hungary Kft.)

^8^Ronapreve® (Roche Registration GmbH )

^9^Valneva® (Valneva Austria GmbH)

^10^Nuvaxovid® (Novavax CZ, a.s.)

^11^Evusheld® (AstraZeneca AB)

^12^Bexsero® (GSK Vaccines S.r.l.)

^13^Vosevi® (Gilead Sciences Ireland UC)

^14^Epclusa® (Gilead Sciences Ireland UC)

^15^Maviret® (AbbVie Deutschland GmbH Co. KG)

^16^Sovaldi® Gilead Sciences Ireland UC)

^17^Biktarvy® (Gilead Sciences Ireland UC)

^18^Stribild® (Gilead Sciences Ireland UC)

^19^Sunlenca® (Gilead Sciences Ireland Unlimited Company)

^20^Harvoni® (Gilead Sciences Ireland UC)

^21^Tybost® (Gilead Sciences Ireland UC)

^22^Viekirax® (AbbVie Deutschland GmbH Co. KG)

^23^Veklury® (Gilead Sciences Ireland UC)

^24^Ervebo® (Merck Sharp & Dohme B.V. )

^25^Apexxnar® ((Pfizer Europe MA EEIG))

^26^Zavicefta® (Pfizer Ireland Pharmaceuticals)

^27^Zabdeno® (Janssen-Cilag International N.V.)

^28^Vepacel® (Ology Bioservices Ireland LTD)

^29^Xofluza® (Roche Registration GmbH)

^30^Mvabea® (Janssen-Cilag International N.V.)

^31^Vaborem® (Menarini International Operations Luxembourg S.A.)

^32^Zerbaxa® (Merck Sharp & Dohme B.V. )

^33^Tivicay® (ViiV Healthcare BV)

^34^Exviera® (AbbVie Ltd)

^35^Supemtek® (Sanofi Pasteur)

^36^Daklinza® (Bristol-Myers Squibb Pharma EEIG)

^37^Triumeq® (ViiV Healthcare B.V.)

^38^Pifeltro® (Merck Sharp & Dohme B.V.)

^39^Delstrigo® (Merck Sharp & Dohme B.V.)

^40^Sivextro® (Merck Sharp & Dohme B.V.)

^41^Olysio® (Janssen-Cilag International NV)

^42^Gardasil 9® (Merck Sharp & Dohme B.V.)

^43^Vitekta® (Gilead Sciences International Ltd)

^44^Prevymis® (Merck Sharp & Dohme B.V.)

^45^Vaxchora® (Emergent Netherlands B.V.)

^46^Fetcroja® (Shionogi B.V.)

^47^Zinforo® (Pfizer Ireland Pharmaceuticals)

^48^Nimenrix® (Pfizer Europe MA EEIG)

^49^Vantobra® (Pari Pharma GmbH)

^50^Xerava® (PAION Deutschland GmbH)

^51^Hepcludex® (Gilead Sciences Ireland UC)

^52^Hexacima® (Sanofi Pasteur)

^53^Sirturo® (Janssen-Cilag International NV)

^54^ Pandemic Influenza Vaccine H5N1 Baxter AG (Ology Bioservices Ireland LTD)

^55^Recarbrio® (Merck Sharp & Dohme B.V.)

^56^Xenleta® (Nabriva Therapeutics Ireland DAC)

^57^Trumenba® (Pfizer Europe MA EEIG)

^58^Deltyba® (Otsuka Novel Products GmbH)

^59^Alpivab® (Biocryst)

^60^Vaxneuvance® (Merck Sharp & Dohme B.V.)

^61^Imvanex® (Bavarian Nordic A/S)

^62^MenQuadfi® (Sanofi Pasteur)

^63^Quofenix® (A. Menarini Industrie Farmaceutiche Riunite s.r.l.)

^64^ Vocabria® (ViiV Healthcare B.V.)

^65^Arikayce liposomal® (Insmed Netherlands B.V.)

^66^Cresemba® (Basilea Pharmaceutica Deutschland GmbH)

^67^Rukobia® (ViiV Healthcare B.V.)

^68^Shingrix® (GlaxoSmithkline Biologicals SA)

^69^Genvoya® (Gilead Sciences International Ltd)

^70^Odefsey® (Gilead Sciences International Ltd)

^71^Zepatier® (Merck Sharp & Dohme B.V.)

^72^Dovprela ® (Mylan Ireland Limited)

^73^Vaxelis® (MCM Vaccine B.V.)

^74^Vemlidy® (Gilead Sciences Ireland UC)

^75^Tecovirimat® SIGA (SIGA Technologies Netherlands B.V.)

^76^Obiltoxaximab SFL® (SFL Pharmaceuticals Deutschland GmbH)

^77^Xydalba® (AbbVie Deutschland GmbH & Co. KG)

^78^Dengvaxia® (Sanofi Pasteur)

^79^Trogarzo® (Theratechnologies Europe Limited)

^80^Heplisav B® (Dynavax GmbH)

^81^Tenkasi® (Menarini International Operations Luxembourg S.A.)

## Table S2. Multivariable model excluding COVID-19 compounds

| **Variable** |  | **Multivariable** | **P** |
| --- | --- | --- | --- |
| **Outbreak setting** | ^a^EID | Ref |  |
|  | Non-EID | -4.1 (-7.8 to -0.4.) | **0.03** |
| **Pathogen(s) targeted** | Virus | Ref |  |
|  | Bacterium | 0.3 (-1.9 to 2.4) | 0.82 |
|  | Other/mix/fungus | 1.8 (-3.8 to 6.8) | 0.48 |
| **Vaccine** | Yes | Ref |  |
|  | No | 0.2 (-2.3 to 2.7) | 0.88 |
| **Combination drug** | No | Ref |  |
|  | Yes | -2.8 (-5.3 to -0.3) | **0.03** |
| **^b^CMA status** | No | Ref |  |
|  | Yes | 3.0 (-2.4 to 8.4) | 0.27 |
| **Orphan drug** | No | Ref |  |
|  | Yes | 0.1 (-4.2 to 4.4) | 0.10 |
| **Accelerated assessment** | No | Ref |  |
|  | Yes | -4.4 (-7.9 to -0.8) | **0.02** |
| **Type of sponsor-investigator of phase 1 trial** | Pharmaceutical | Ref |  |
|  | Academic | -0.5 (-9.0 to 8.0) | 0.79 |
|  | Governmental | 3.7 (-1.0 to 8.4) | 0.19 |

^a^EID: emerging infectious disease. ^b^CMA: conditional marketing authorization. CI: 95% confidence interval; Ref, reference category.

## Table S3. Median clinical development times for FDA approved compounds per disease targeted, in years (median, IQR) compared to EMA

| Type of disease targeted | N | Clinical development time (median (IQR))  FDA | EMA |
| --- | --- | --- | --- |
| COVID-19 | 8 | 0.8 (0.6 – 1.8) | 1.1 (0.7-1.5) |
| Hepatitis (A-E) | 11 | 5.0 (3.8-7.7) | 4.5 (3.7-7.3) |
| Ebola virus disease | 1 | 5.2 (5.2 – 5.2) | 5.1 (5.1-5.1) |
| Influenza | 2 | 6.2 (3.6 – 8.8) | 9.0 (5.8-12.1) |
| HIV | 15 | 6.9 (5.4 – 13.4) | 7.1 (4.4-13.7) |
| Other bacterial disease^a^ | 21 | 8.7 (5.3 – 10.3) | 9.0 ( 7.2-12.6) |
| DR-TB | 2 | 11.0 (8.5-13.5) | 12.1 (9.7-14.4) |
| Other^b^ | 8 | 12.6 (9.6 – 17.4) | 13.1 (10.1-14.9) |
|  |  |  |  |
| Overall | 68 | 7.0 (4.2 – 11.9) | 7.8 (4.4 – 12.9) |

Note. IQR, interquartile range; DR-TB, drug-resistant tuberculosis

^a^Including vaccines targeting *meningococci, pneumococci,* and *Vibrio cholerae,* and antibiotics or monoclonal antibodies targeting Gram-negative bacterial infections, respiratory tract infections, community acquired bacterial infections, intra-abdominal infections, and anthrax)

**^b^**Including vaccines targeting human papilloma virus, a combination of diphtheria/acellular pertussis/tetanus/polio/haemophilus influenzae b/hepatitis B, poxviridae infections, herpes zoster, and dengue, and antivirals targeting cytomegalovirus, poxviridae infections, and antimycotics targeting aspergillosis

## Table S4: STROBE check-list

|  | Item No | Recommendation |  | Manuscript location |
| --- | --- | --- | --- | --- |
| **Title and abstract** | 1 | (*a*) Indicate the study’s design with a commonly used term in the title or the abstract |  | Page 1 |
|  |  | (*b*) Provide in the abstract an informative and balanced summary of what was done and what was found |  | Page 3, 4 |
| Introduction | | |  |  |
| Background/rationale | 2 | Explain the scientific background and rationale for the investigation being reported |  | Page 7, 8 |
| Objectives | 3 | State specific objectives, including any prespecified hypotheses |  | Page 8 |
| Methods | | |  |  |
| Study design | 4 | Present key elements of study design early in the paper |  | Page 8, 9 |
| Setting | 5 | Describe the setting, locations, and relevant dates, including periods of recruitment, exposure, follow-up, and data collection |  | Page 9 - 11 |
| Participants | 6 | (*a*) Give the eligibility criteria, and the sources and methods of selection of participants |  | Page 9 - 11 |
| Variables | 7 | Clearly define all outcomes, exposures, predictors, potential confounders, and effect modifiers. Give diagnostic criteria, if applicable |  | Page 11, 12 |
| Data sources/ measurement | 8* | For each variable of interest, give sources of data and details of methods of assessment (measurement). Describe comparability of assessment methods if there is more than one group |  | Page 9 - 11 |
| Bias | 9 | Describe any efforts to address potential sources of bias |  | Page 8 -11, 24 |
| Study size | 10 | Explain how the study size was arrived at |  | NA |
| Quantitative variables | 11 | Explain how quantitative variables were handled in the analyses. If applicable, describe which groupings were chosen and why |  | Page 12, 13 |
| Statistical methods | 12 | (*a*) Describe all statistical methods, including those used to control for confounding |  | Page 12, 13 |
|  |  | (*b*) Describe any methods used to examine subgroups and interactions |  | Page 12 |
|  |  | (*c*) Explain how missing data were addressed |  | Page 10, 11 |
|  |  | (*d*) If applicable, describe analytical methods taking account of sampling strategy |  | NA |
|  |  | (*e*) Describe any sensitivity analyses |  | Page 13 |
| Results | | |  |  |
| Participants | 13* | (a) Report numbers of individuals at each stage of study—eg numbers potentially eligible, examined for eligibility, confirmed eligible, included in the study, completing follow-up, and analysed |  | Page 13, 14 |
|  |  | (b) Give reasons for non-participation at each stage |  | Page 13, 14 |
|  |  | (c) Consider use of a flow diagram |  | Page 13, Fig. 2 |
| Descriptive data | 14* | (a) Give characteristics of study participants (eg demographic, clinical, social) and information on exposures and potential confounders |  | Page 14 |
|  |  | (b) Indicate number of participants with missing data for each variable of interest |  | NA |
| Outcome data | 15* | Report numbers of outcome events or summary measures |  | Page 14-16 |
| Main results | 16 | (*a*) Give unadjusted estimates and, if applicable, confounder-adjusted estimates and their precision (eg, 95% confidence interval). Make clear which confounders were adjusted for and why they were included |  | Page 16, 17 |
|  |  | (*b*) Report category boundaries when continuous variables were categorized |  | NA |
|  |  | (*c*) If relevant, consider translating estimates of relative risk into absolute risk for a meaningful time period |  | NA |
| Other analyses | 17 | Report other analyses done—eg analyses of subgroups and interactions, and sensitivity analyses |  | Page 16, 17, Supplementary file |
| Discussion | | |  |  |
| Key results | 18 | Summarise key results with reference to study objectives |  | Page 17 |
| Limitations | 19 | Discuss limitations of the study, taking into account sources of potential bias or imprecision. Discuss both direction and magnitude of any potential bias |  | Page 24, 25 |
| Interpretation | 20 | Give a cautious overall interpretation of results considering objectives, limitations, multiplicity of analyses, results from similar studies, and other relevant evidence |  | Page 17 - 23 |
| Generalisability | 21 | Discuss the generalisability (external validity) of the study results |  | Page 25, 26 |
| Other information | | |  |  |
| Funding | 22 | Give the source of funding and the role of the funders for the present study and, if applicable, for the original study on which the present article is based |  | Page 28 |

*Give information separately for exposed and unexposed groups.

**Note:** An Explanation and Elaboration article discusses each checklist item and gives methodological background and published examples of transparent reporting. The STROBE checklist is best used in conjunction with this article (freely available on the Web sites of PLoS Medicine at http://www.plosmedicine.org/, Annals of Internal Medicine at http://www.annals.org/, and Epidemiology at http://www.epidem.com/). Information on the STROBE Initiative is available at www.strobe-statement.org.
